# Editorial: Oncolytic virotherapy, volume II

**DOI:** 10.3389/fmolb.2025.1552643

**Published:** 2025-01-21

**Authors:** Ahmed Majeed Al-Shammari, Pier Paolo Piccaluga

**Affiliations:** ^1^ Experimental Therapy Department, Iraqi Center for Cancer and Medical Genetic Research, Mustansiriyah University, Baghdad, Iraq; ^2^ Biobank of Research, IRCCS Azienda Ospedaliera-Universitaria di Bologna Policlinico di S. Orsola, Bologna, Italy; ^3^ Department of Medical and Surgical Sciences, Bologna University School of Medicine, Bologna, Italy

**Keywords:** T-VEC, HSV-1, NDV, VSV, oncolytic virus

## 1 Introduction and background

**FIGURE 1:**
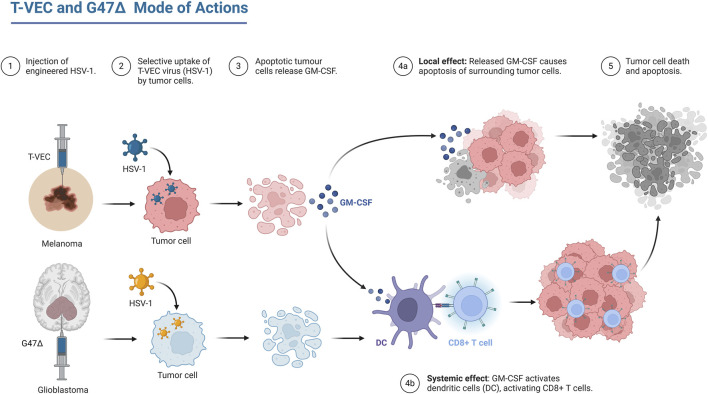
Mechanism of action of two of oncolytic HSV, T-Vec which is an FDA approved therapy and G47∆ which is phase II clinical trials. Created in BioRender. (Andtbacka et al., 2015) https://BioRender.com/b44g764.

Oncolytic Virotherapy (OV) is continuing to evolve rapidly, with one oncolytic herpes simplex virus (HSV) clinically approved oncolytic virotherapeutic, by FDA named Talimogene laherparepvec (T-VEC) to treat Advanced Melanoma (Andtbacka et al., 2015), and with many other viruses in clinical trials, one of them named G47∆ which is also HSV in phase 2 clinical trial to treat residual or recurrent glioblastoma (Todo et al., 2022). The main advantage of OV is its direct tumor cell killing and its ability to stimulate specific immune response against the cancer cells as shown in Figure 1.

In the current Research Topic, seven articles were submitted that focus about oncolytic virotherapy, including two original research articles, three reviews, one brief research article, and one case report. Four of these articles discuss the HSV as a promising oncolytic virus that targets cancer cells selectively and as oncolytic immunotherapy. This reflects the importance of this virus in the Oncolytic virotherapeutic field mainly due to the two clinically approved OV therapies. The Research Topic articles were categorized into two main sections: 1) The promising use of HSV as oncolytic virus; 2) NDV, VSV and other oncolytic viruses in clinical and preclinical experiments.

### 1.1 The promising use of herpes simplex virus (HSV) as oncolytic virus

In a review by Li et al. and his group focuses about the potential of oncolytic herpes simplex virus (HSV) as specific therapy to digestive system tumors (DSTs) which are a significant global health burden. HSV, with its large genome and capacity for genetic modification, can directly lyse tumor cells and stimulate anti-tumor immune responses. Preclinical and clinical studies have shown the efficacy of HSV-based oncolytic virotherapies in various DSTs, including esophageal, gastric, pancreatic, and liver cancers. HSV has also shown potential as a diagnostic tool for detecting circulating tumor cells in pancreatic cancer. It is also highlighted that HSV can directly lyse tumor cells and stimulate anti-tumor immune responses. Continued research and development are crucial to optimize HSV-based therapies, including developing more targeted and potent oncolytic viruses and exploring combination therapies with other modalities.

A study by Vannini et al. and his colleagues describes the development and characterization of R-421, a novel oncolytic herpesvirus specifically targeted to nectin4, a recently discovered tumor-associated antigen overexpressed in several cancers. R-421 was engineered by replacing the scFv to HER2 with the scFv directed against nectin4. Importantly, R-421 was detargeted from the natural herpesvirus receptors, nectin1 and HVEM, ensuring strict dependence on nectin4 for entry. R-421 is a derivative of the HER2-retargeted prototype ReHV named R-337. They conducted *in vitro* study in human cancer cell lines expressing high levels of nectin4, they found that R-421 efficiently infected and lysed these cancer cells while sparing cells with low or moderate nectin4 expression, including normal human fibroblasts. They also identified a threshold level of nectin4 expression for R-421 infection lies in between 11K and 5K MeFI values, below which cells were resistant to R-421 infection, regardless of their malignant or non-malignant origin. Moreover, they tested the new virus *in vivo*, and found to effectively inhibited the growth of murine tumors engineered to express human nectin4. Furthermore, R-421 demonstrated potent anti-tumor activity in combination with immune checkpoint inhibitors and was augmented by the immunomodulator cyclophosphamide. The anti-tumor efficacy of R-421/αPD1 combination is T cell-mediated and dependent on CD8-positive lymphocytes. Markedly, R-421 induced long-lasting anti-tumor immunity, providing protection against subsequent challenges with the same tumor cells. These results show the potential of R-421 as a novel oncolytic virotherapy for nectin4-positive cancers, including difficult to treat cancers such as triple-negative breast cancer, and pancreatic ductal carcinoma. The strict dependence on nectin4 expression for infection and the observed safety profile suggest that R-421 may offer a targeted and potentially less toxic therapeutic option for these challenging cancers.

Robilotti et al. and his co-authors discussed the oncolytic viral immunotherapies (OVIs) like Talimogene laherparepvec (T-VEC) which is the only OV approved for patient use in the United States. T-VEC is a modified herpes simplex virus type 1 (HSV-1), show promise in cancer treatment. However, current biosafety guidelines often treated T-VEC with the same of those for wild-type HSV-1, leading to excessively restrictive handling practices. This review argues that T-VEC’s safety profile and limited shedding and infectivity and low risk of spread to healthcare workers warrant less stringent measures, such as BSL-1 precautions. Updated guidelines could improve patient access and reduce logistical burdens for treatment centers.

Nabi et al. presented a study on the oncolytic herpes simplex virus type-1 (HSV-1) vaccine strain VC2, which demonstrated an interesting potential in treating advanced breast cancer, particularly in a highly metastatic murine model (2). The authors highlight the efficacy of VC2 in inducing immune responses and reducing tumor metastasis in the 4T1/Balb/c mouse model of stage four breast cancer. In their investigation, they found that while VC2 treatment did not significantly decrease the size of primary tumors, it led to a marked reduction in lung metastases, which was associated with increased infiltration of functionally active T cells, specifically CD4^+^ and CD4^+^CD8^+^ double-positive T cells, into the tumors (2). Furthermore, the study elaborates on the mechanism by which VC2 operates, emphasizing its ability to replicate within cancer cells while sparing normal cells due to engineered modifications that prevent entry into neuronal tissues. This selective replication not only leads to tumor lysis but also enhances the presentation of tumor neoantigens, which in turn stimulates a robust immune response. The authors note that the presence of tumor-infiltrating lymphocytes correlates with better prognosis, underscoring the significance of T cell infiltration in controlling cancer progression. Notably, the research highlights the downregulation of pro-tumor markers, such as PD-L1 and VEGF, in metastatic lesions following VC2 treatment. This suggests that not only does VC2 therapy drive an anti-tumor immune response, but it also alters the tumor microenvironment in a way that is unfavorable for cancer progression. The authors argue that these findings support the potential of VC2 as an effective oncolytic and immunotherapeutic approach for treating breast cancer and potentially other malignancies.

### 1.2 NDV, VSV and other oncolytic viruses in clinical and preclinical experiments

Oncolytic viruses (OVs) have garnered significant attention as a novel approach against different cancers, by selectively targeting and killing tumor cells while sparing normal cells. IL-12, a pro-inflammatory cytokine, plays a crucial role in enhancing the immune response against tumors by stimulating the activation of T cells and natural killer (NK) cells, thus promoting anti-tumor immunity. The study presented by Abdulal et al. investigated the construction and potential therapeutic applications of a genetically modified oncolytic virus, VSVΔ51M, designed to express human interleukin-12 (IL-12) for cancer treatment (1). Particularly, in their research, the authors constructed the VSVΔ51M virus by modifying the vesicular stomatitis virus (VSV) backbone, by eliminating the methionine residue at position 51 in the matrix protein to reduce its cytotoxicity while retaining its oncolytic properties. The study emphasizes the importance of localizing IL-12 expression within tumors to mitigate systemic toxicity associated with traditional cancer therapies. The engineered virus, VSVΔ51M-hIL-12, was shown to express IL-12 effectively, leading to heightened immune cell activation and subsequent tumor cell apoptosis (1). Preclinical data confirmed the safety profile and limited toxicity of VSVΔ51M-hIL-12, with *in vitro* experiments demonstrating its ability to induce significant cytotoxic effects against various cancer cell lines, including MCF-7 and B16F10. The authors further elucidated the mechanisms underlying the virus’s oncolytic efficacy by examining viral replication kinetics and immune responses. Notably, co-culture experiments with peripheral blood mononuclear cells (PBMCs) revealed that VSVΔ51M-hIL-12 not only enhanced the production of IFN-γ but also activated NK cells, underscoring the therapeutic potential of combining IL-12 with oncolytic virotherapy. In addition, *in vivo* studies using a murine model of melanoma provided compelling evidence for the antitumor efficacy of VSVΔ51M-hIL-12, demonstrating significant delays in tumor progression and prolonged survival compared to untreated controls. The findings suggest that the VSVΔ51M platform, particularly with the incorporation of hIL-12, represents a promising strategy for enhancing the immune response against cancer, warranting further investigation and potential clinical development.

In a study authored by Al-Shammari and Salman, explores the antimetastatic and anticancer activities of the Newcastle Disease Virus (NDV) AMHA1 in a 3D spheroid model of breast cancer metastasis. NDV can kill cancer cells through direct replication or induction of apoptosis. They used two breast cancer cell lines, AMJ13 and MCF-7, were used to create a metastasis model. The study evaluated the ability of NDV AMHA1 to infect and kill these cells both in 2D and 3D culture systems. Results showed that NDV AMHA1 effectively infected and killed breast cancer cells in both 2D adherent cultures and 3D spheroids at various virus doses. It showed significant cell death, especially in the AMJ13 spheroids. Furthermore, NDV induced more than 80% oncolysis within 24 h of infection in both AMJ13 and MCF-7 cells, with continued effects up to 5 days. Moreover, NDV significantly inhibited the reattachment and growth of breast cancer spheroids, indicating its potential to prevent metastasis. NDV AMHA1 induced apoptosis in cancer spheroids, evidenced by higher expression of caspase-3 and P21, and reduced expression of the proliferation marker Ki67. NDV AMHA1 has a potent oncolytic effect against breast cancer cells and metastasis models, working through the induction of apoptosis and downregulation of key proliferation markers. The virus selectively targets cancer cells without harming normal cells, making it a promising candidate for clinical breast cancer therapy. The study concluded that NDV AMHA1 shows significant antitumor and antimetastatic activities in breast cancer models, suggesting its potential as an effective therapy for metastatic breast cancer.

Finally, the case report presented by Gesundheit et al. explores the efficacy of intratumoral oncolytic virotherapy (IT-OV) in the treatment of metastatic breast cancer in a 40-year-old Caucasian female patient (3). The patient presented with a grade-3 hormone receptor-positive and HER2-positive breast tumor, alongside two lung metastatic nodules. Following her refusal of aggressive standard treatments, the patient was administered a combination of intramuscular trastuzumab, oral tamoxifen, and customized IT-OV over a 17-month period, incorporating various oncolytic viruses (OVs) such as Newcastle disease virus, reovirus, and vaccinia virus. Initial PET/CT imaging after three and 6 months indicated an increase in tumor size and metabolic activity, which was interpreted as a hyperimmune response rather than disease progression. The subsequent months revealed a significant decline in both tumor size and metabolic activity, ultimately leading to the absence of radiographic evidence of disease. This observation suggests a potential abscopal effect, wherein localized therapeutic responses to IT-OV triggered systemic immune activation, leading to the regression of distant metastatic lesions. The treatment was well tolerated, and the patient maintained a good quality of life throughout the intervention. The authors emphasize that this case underscores the promising role of IT-OV as an adjunctive therapy in metastatic breast cancer management, particularly in patients with limited treatment options. They argue for the necessity of further clinical studies to validate these findings and optimize the therapeutic regimens involving oncolytic viruses, particularly in the context of immunotherapy (3).

## 2 Conclusion

Overall, these studies presented a compelling argument for the integration of innovative virotherapeutic approaches in the treatment paradigms for advanced cancer, with implications and new perspectives for future clinical research. It is expected that oncolytic virotherapy will be as first line therapy for advanced cancers in near future especially after approving being safe and effective
